# Gut Microbiota as a Source of Uremic Toxins

**DOI:** 10.3390/ijms23010483

**Published:** 2022-01-01

**Authors:** Vasily A. Popkov, Anastasia A. Zharikova, Evgenia A. Demchenko, Nadezda V. Andrianova, Dmitry B. Zorov, Egor Y. Plotnikov

**Affiliations:** 1 A.N. Belozersky Institute of Physico-Chemical Biology, Lomonosov Moscow State University, 119992 Moscow, Russia; popkov@belozersky.msu.ru (V.A.P.); azharikova89@gmail.com (A.A.Z.); demchenko.jane@mail.ru (E.A.D.); andnadya12@gmail.com (N.V.A.); zorov@genebee.msu.ru (D.B.Z.); 2 V.I. Kulakov National Medical Research Center of Obstetrics, Gynecology and Perinatology, 117997 Moscow, Russia; 3 Faculty of Bioengineering and Bioinformatics, Lomonosov Moscow State University, 119992 Moscow, Russia

**Keywords:** uremia, uremic toxins, microbiome, chronic kidney disease

## Abstract

Uremic retention solutes are the compounds that accumulate in the blood when kidney excretory function is impaired. Some of these compounds are toxic at high concentrations and are usually known as “uremic toxins”. The cumulative detrimental effect of uremic toxins results in numerous health problems and eventually mortality during acute or chronic uremia, especially in end-stage renal disease. More than 100 different solutes increase during uremia; however, the exact origin for most of them is still debatable. There are three main sources for such compounds: exogenous ones are consumed with food, whereas endogenous ones are produced by the host metabolism or by symbiotic microbiota metabolism. In this article, we identify uremic retention solutes presumably of gut microbiota origin. We used database analysis to obtain data on the enzymatic reactions in bacteria and human organisms that potentially yield uremic retention solutes and hence to determine what toxins could be synthesized in bacteria residing in the human gut. We selected biochemical pathways resulting in uremic retention solutes synthesis related to specific bacterial strains and revealed links between toxin concentration in uremia and the proportion of different bacteria species which can synthesize the toxin. The detected bacterial species essential for the synthesis of uremic retention solutes were then verified using the Human Microbiome Project database. Moreover, we defined the relative abundance of human toxin-generating enzymes as well as the possibility of the synthesis of a particular toxin by the human metabolism. Our study presents a novel bioinformatics approach for the elucidation of the origin of both uremic retention solutes and uremic toxins and for searching for the most likely human microbiome producers of toxins that can be targeted and used for the therapy of adverse consequences of uremia.

## 1. Introduction

Chronic kidney disease (CKD) is a common health problem in adults defined as a gradual loss in kidney function. It affects about 10% of the human population worldwide [1]. CKD is characterized by reduced glomerular filtration rate, enhanced urinary albumin excretion [2], and accumulation of many metabolic waste products in the organism that are normally excreted, predominantly by the kidneys. These metabolites are called uremic retention solutes or uremic toxins in the case of toxicity at uremic concentrations [3]. Their accumulation causes a great number of pathologies which are collectively named uremic syndrome or uremia [4]. The complications of uremia include multi-organ dysfunctions such as bone diseases, serositis, insulin resistance, renal fibrosis, podocyte dysfunction, decreased mental acuity, and various cardiovascular problems [4,5]. Some of these ailments are present in the World Health Organization list of widespread causes of death. Despite the severity of the consequences of uremia and despite intense study of the topic, the cellular and molecular mechanisms underlying syndrome development mostly remain unclear. The main reason for this is the huge and continuously expanding list of uremic retention solutes [6], which complicates the analysis of the impact of each of them and the elucidation of the possible interplays between them. Nowadays, the European Uremic Solutes Database (EUTox-DB), which was created by The European Uremic Toxin Work Group (EUTox), contains 130 solutes [7,8,9,10]. The substances belong to different chemical classes and participate in a great diversity of biochemical pathways, making further classification difficult.

Considering that the human gut metagenome contains 150 times more genes than the host [11], it is not surprising that intestinal bacteria produce a huge variety of unique substances, some of which could be related to uremic toxins. Indeed, there are some observations that the gut microbiota contributes to the uremic toxins production that inevitably aggravates the health status of CKD patients. Thus, as early as 1966, Einheber and Carter removed kidneys from germ-free rats and rats with normal microflora, thus creating rats that could not excrete uremic toxins and hence died due to uremia. Remarkably, the germ-free animals remained alive significantly longer than those with the normal gut microbiome [12]. Comparison of mural plasma from germ-free and conventional-microbiota rats revealed the emergence of many uremic retention solutes including indole-3-propionic acid, indoxyl sulfate, and p-cresyl sulfate in the conventional-microbiota rats after nephrectomy, which were less abundant in the germ-free animals [13]. Similarly, the microbiota was found to be important in the health/disease balance under renal failure in humans also. It was shown that hemodialysis patients who underwent a colon resection demonstrated lower levels of some uremic retention solutes and that dialysis patients with intact colons accumulated more than 30 additional substances in plasma. Those substances were assigned to the group of gut-derived uremic solutes [14].

Today, the list of microbiota-derived toxins is growing. The bacterial origin of substances such as p-cresyl sulfate, indoxyl sulfate, indole-3-acetic acid, trimethylamine, trimethylamine-N-oxide, hippuric acid, phenol, and phenylacetic acid has been proved in several independent experiments [15,16,17,18]. However, most compounds in the EUTox-DB are underexplored. According to the Human Metabolome Database, 69 uremic retention solutes are classified as endogenous, and 56 have not yet been classified [14,19,20,21,22].

In this study, we analyzed the Human Metabolome, Kyoto Encyclopedia of Genes and Genomes (KEGG), MetaCyc, and Human Protein Atlas databases to find microbial biochemical pathways and enzymes strongly associated with uremic toxin synthesis.

## 2. Results

We used a list of 142 substances regarded as uremic retention solutes for the analysis. These were the 130 solutes described in the EUTox database and an additional 12 compounds referred to in publications [15,23,24] as potential uremic toxins. Among them, 54 compounds were found in the KEGG database as products of, or participants in, some biochemical reactions, and these uremic retention solutes were included in further analysis.

Using data from KEGG, we assigned each toxin to a specific enzymatic reaction as a product or substrate. Then, using the NIH Human Microbiome Project database, all the bacteria in the human microbiome were identified. Lastly, all the metabolic pathways for these bacteria were found in KEGG. As a result, we obtained a complete list of the toxins with enzymatic pathways found in the bacteria of the human microbiome that could produce these reactions.

Full data on bacterial synthetic and degrading enzymatic reactions for uremic retention solutes are available in the Supplementary Table S1, with detailed data on the bacterial strains and KEGG reactions involved, including 186,186 toxin-reaction–bacteria links. These data were then subjected to a more in-depth analysis, as presented below.

### 2.1. Toxins That Can Be Synthesized by the Microbiome

In Table 1, we summarize the data on toxins associated with the human gut microbiome: the number of bacteria from the gut microbiome that have reactions described in KEGG for the synthesis or metabolism of a given toxin, the number of different reactions for the given toxin in bacteria, and the number of KEGG-described enzymatic reactions for the given toxin in the human organism.

Based on the KEGG database, we showed that only eight uremic retention solutes had no attributed pathways in human metabolism but had ascribed enzymes in bacteria (Table 1, and extended version in Supplementary Table S2). These compounds are mannitol, phenol, trimethylamine, oxalate, creatinine, trimethylamine-N-oxide, pseudouridine, and 3-(3-hydroxyphenyl) propanoic acid. It should be noted that our analysis included only enzymatic reactions described in KEGG, and thus, compounds such as creatinine or oxalate that can be produced non-enzymatically in human organisms fall into this category. Other important exceptions in this table are trimethylamine and trimethylamine-N-oxide, since there is an enzyme (flavin monooxygenase) in the human liver that determines the conversion of trimethylamine into trimethylamine-N-oxide [25]. However, due to the limitations of the KEGG database, our analysis yielded zero enzymes for these uremic toxins.

We also tested whether the number of potential toxin-synthesizing bacteria correlated with the toxin concentrations in either healthy or uremic conditions. However, the correlation coefficients for both conditions were non-significant and near-zero, and thus no strong correlation was observed between toxin concentration and the number of bacteria producing it.

### 2.2. Toxins with the Least Abundant Synthesizing Human Enzymes

Besides uremic retention solutes, which have no annotated synthesizing enzymes in *Homo sapiens*, we found 33 toxins for which a few enzymes (from one to three enzymes) could be assigned as synthetic in the human organism (Table 1). To test whether these enzymes result in a meaningful production of given toxins in humans, we evaluated the amount of these enzymes in the human body.

**Figure 1:**
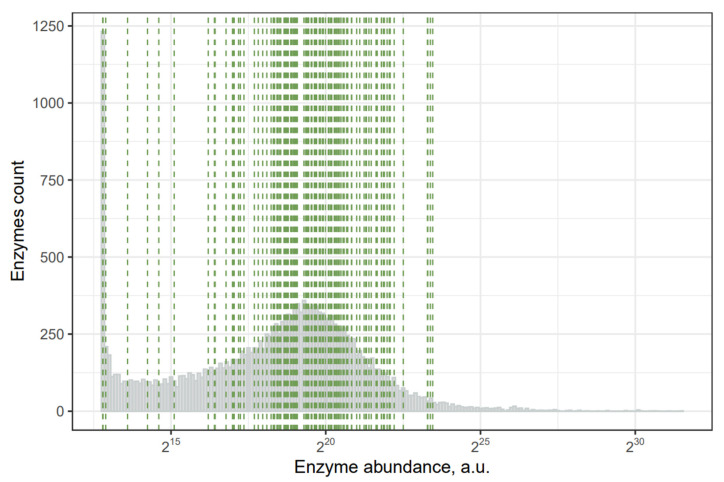
Abundance of human mRNA expression in the whole organism: green lines—genes of uremic-retention-solutes-synthesizing enzymes.

From the Human Protein Atlas database, we extracted the mRNA abundance data in different tissues and normalized it with respect to average tissue weight. We obtained an approximate abundance for particular enzymes in the human organism using this approach. All human gene expression abundance in the whole organism demonstrates a bimodal distribution, with a large portion of genes being poorly represented and others showing an almost normal distribution. In Figure 1, green dashes indicate the position of uremic-retention-solutes-synthesizing enzymes. According to our analysis, the most abundant enzymes are responsible for synthesizing creatine, indole-3-acetic acid, nicotinamide, methylglyoxal, and S-adenosylhomocysteine. Thus, these toxins are expected to be predominantly produced by the human organism. The five least abundant enzymes are responsible for the synthesis of melatonin, hexanal, orotidine, a-keto-d-guanidinovaleric acid, and 3-hydroxyanthranilic acid. Based on these results, we suggest that the human metabolism might play an insignificant role in producing these five compounds and that they are mainly produced by the microbiome.

### 2.3. Bacteria with the Ability to Synthesize or Metabolize Uremic Retention Solutes

Data from KEGG also allowed us to address the questions about the various bacteria’s ability to synthesize uremic retention solutes. Using KEGG, it was possible to extract data on whether a toxin is “upstream” or “downstream” in the specific pathway in a reaction, which theoretically should correlate with the toxin being synthesized or metabolized in a given reaction. We summed the number of toxins that certain bacteria can potentially synthesize or metabolize, using the KEGG data. Complete data for 142 bacteria taxa are available in Supplementary Table S3. From this list, 70 bacteria can potentially synthesize more than 20 uremic retention solutes, while only 20 species can metabolize a similar number of solutes.

We also estimated how many toxins gut bacteria can synthesize without being able to metabolize the same toxin, and vice versa. Using this approach, we discovered that *Brevundimonas* sp., *Campylobacter coli*, *Desulfovibrio* sp., *Oxalobacter formigenes*, *Campylobacter upsaliensis*, *Helicobacter pylori*, *Phascolarctobacterium faecium*, and *Desulfovibrio piger* can synthesize more than 14 toxins without being able to metabolize the same toxins.

In contrast, the following bacteria can only metabolize more than seven toxins without being able to synthesize them: *Pediococcus acidilactici*, *Listeria innocua*, *Listeria grayi*, *Lactobacillus ruminis*, *Klebsiella oxytoca*, *Clostridium sporogenes*, *Escherichia coli*, *Klebsiella* sp., *Enterococcus faecalis*, *Ruminococcaceae bacterium*, and *Parvimonas micra*.

However, in general, the reaction direction of KEGG pathways is not strictly determined. Some KEGG reactions are included in separated “modules”, where the reaction direction is given. Sadly, only 46 of 336 reactions are included in KEGG modules. We added information from the MetaCyc database, which contains more detailed information on reaction directions. Combining these two databases, we were able to strictly determine the direction of 151 of 336 enzymatic reactions that are responsible for toxin synthesis/metabolism. Using this approach, we could more accurately identify the ability of bacteria to synthesize (without decomposing) or decompose (without synthesizing) individual toxins. The most significant taxons of synthesizing and decomposing bacteria are given in Table 2; a complete list is available in Supplementary Table S3. Note that several bacterial taxa were included both in the list of toxin producers and in the list of toxin consumers. This apparent contradiction is explained by the fact that some species (genera) of bacteria can synthesize certain toxins but at the same time consume (that is, remove) some other toxins. For example, *Pediococcus acidilactici* has metabolic pathways for the synthesis of homocysteine, indole-3-acetic acid, myoinositol, nicotinamide, and y-guanidinobutyric acid and at the same time is able to consume creatinine, cytidine, methylglyoxal, S-adenosylhomocysteine, sorbitol, and xanthosine. Specific uremic retention solutes synthesized/decomposed by a particular bacterium can be found in Supplementary Table S4.

### 2.4. Human Microbiome Project metadata analysis

To check the data obtained from KEGG and bacterial genomes, we used the Human Microbiome Project (HMP2) database, which contains transcriptomic and proteomic data for the microbiota of 735 people. Using this approach, we determined which bacteria are present in the microbiome of what percentage of HMP2 patients, what mRNA of the uremic-retention-solutes-synthesizing enzymes are detected in the metatranscriptome, and for which of them the protein was detected in the proteome. The complete dataset can be seen in Supplementary Table S5.

Among uremic retention solutes, 46 had the mRNA of at least one potentially synthesizing enzyme detected in at least one patient’s microbiome. Twenty-four of them had at least one enzyme’s mRNA detected in 75% of all patients (Table 3). Thirteen toxins had at least one enzyme detected in less than 10% of patients, and these were: trimethylamine-N-oxide, pseudouridine, p-cresyl sulfate, 3-(3-hydroxyphenyl) propanoic acid, a-keto-d-guanidinovaleric acid, indole-3-acetic acid, y-guanidinobutyric acid, hyaluronic acid (hyaluronan), gentisic acid, kinurenine, dimethylamine, kynurenic acid, asymmetric dimethylarginine (ADMA), and taurocyamine.

We used KEGG data to determine which reactions could potentially yield uremic retention solutes and, using the EC code for enzymes, we determined the enzymes in the metatranscriptome that might be involved in uremic retention solutes synthesis (Table 4). For each of 173 such uremic-retention-solutes-synthesizing enzymes detected in the metatranscriptome, there was a calculated ratio of patients who had mRNA or protein detected in the microbiome samples. We analyzed the mRNA count to determine the abundance of each mRNA type. We ascribed all enzymes to quartiles according to the distribution of all mRNAs, which roughly described the abundances of mRNA in the metatranscriptome. The mRNAs for 64 uremic-retention-solutes-synthesizing enzymes were detected in more than 50% of patients, but only 9 of them had a protein detected in more than 20% of patients. A total of 21 enzymes’ mRNAs were ascribed to more than 100 different bacteria species, while 96 of them were ascribed to fewer than 10. The most common enzymes were associated with the synthesis of arginic acid, orotidine, hypoxanthine, inosine, nicotinamide, xanthine, xanthosine, methylglyoxal, and homocysteine.

Finally, we checked in what percentage of patients each bacterium was present and for how many toxins they expressed the mRNAs of enzymes (Table 5). A total of 313 bacteria species were detected in this database. The top 20 bacteria by frequency of occurrence in patients are listed in Table 5, and the full list can be found in Supplementary Table S5. Of these, 27 bacteria were present in more than 50% of patients and 93 in less than 10%. It should be noted that the abundance of bacteria increases the possibility of mRNA detection. Thus, rare bacteria have fewer “uremic enzymes” detected, but this can be explained by the detection threshold. The most common bacteria were *Ruminococcus torques*, *Faecalibacterium prausnitzii*, *Eubacterium rectale*, *Bacteroides uniformis*, *Bacteroides ovatus*, *Bacteroides vulgatus*, and *Ruminococcus obeum*. *Escherichia coli* and *Klebsiella pneumoniae* could synthesize the highest number of different toxins, at 31 and 30, respectively.

## 3. Discussion

The main goal of this study was to undertake a bioinformatic analysis of the possible contribution of the intestinal microbiota to the synthesis of uremic toxins and the development of uremia in renal failure conditions. To date, several experimental studies have demonstrated the possibility of such a link. In a recent study by Gryp et al. [26], bacteria that might synthesize uremic toxins and/or their precursors (p-cresyl sulfate, indoxyl sulfate, indole-3-acetic acid) were isolated and analyzed. The study revealed that some bacteria can release uremic toxins and/or precursors to the culture medium. These results correlate with our findings. For example, *Lachnospiraceae*, found by Gryp et al. to be one of the uremic-toxins-synthesizing bacteria, is prominently present in our genomic and transcriptomic analysis. The excellent study mentioned here highlighted some points that indicated the relevance of our bioinformatic analysis. As noted by Gryp et al., there was no direct correlation between bacteria with known capacities to synthesize uremic solutes and CKD-provoked microbiota patterns (although there was some correlation for dialysis patients). Note that this type of research requires complex protocol optimization for specific bacteria to be cultivated. Among all microbiota bacteria species, only a small fraction of gut bacterial species can be cultivated, and thus, many bacteria will not be checked in vitro for uremic toxins production. Studies focusing on clinical data from CKD patients have one fundamental limitation: they focus only on bacteria that change (increase or decrease) during CKD. However, these bacteria can produce uremic toxins, use them as substrates, or benefit/suffer from CKD-associated conditions not directly related to kidney dysfunction. Indeed, toxin-producing bacteria do not score significantly higher in CKD patients, as Gryp et al. found. For this reason, we focused on the theoretical capacity of bacteria to produce uremic toxins without focusing on their association with CKD or their abundance, thus bypassing the indicated limitations. We believe that such an approach can help find promising new targets (both bacteria and enzymes) for analysis, which might otherwise be hidden “beneath” major CKD-affected bacteria species.

We collected data on the potential for production of uremic retention solutes for each bacterium that could be found in the microbiome. The goal of this analysis was to identify “bad” and “good” bacteria in terms of uremic toxin production, i.e., to present putative targets for therapy of uremia. Of course, the fact that gut bacteria can potentially synthesize the toxin does not mean that the toxin inevitably enters the bloodstream since bacteria (the same or others) can consume it. Therefore, we also included the ability to metabolize toxins in our analysis. Predictably, the bacteria were the same as those synthesizing the toxin in most cases since the identified solute was just an intermediate in specific metabolic pathways. Theoretically, it is also possible to determine what bacteria can decompose some toxins without being able to synthesize them and, vice versa, what bacteria can synthesize certain toxins but do not have downstream metabolizing enzymes. Available databases do not include enough information for a full-scale analysis of this type.

Nevertheless, our approach still gives some promising results. We defined *Desulfovibrio piger* as a potentially “bad” bacterium, synthesizing more toxins than it can degrade. Aronov and colleagues showed that *Desulfovibrio piger* is associated with a bad prognosis for uremia [14]. However, in the HMP2 database, *Desulfovibrio piger* was detected only in 5% of people, while it expresses enzymes for the potential synthesis of up to 18 toxins. *Eggerthella lenta* was also a “bad”, toxin-synthesizing bacterium in our analysis, and in parallel with our study it was shown that this bacteria is associated with increased production of uremic toxins [27]. *Eggerthella lenta* was even less abundant in HMP2 patients: it was present only in 1.2% of people. Another “bad” bacterium *Campylobacter upsaliensis*, which was one of the most prominent “toxin-synthesizing bacteria” in our analysis, has been associated with uremic syndrome [28]. In a case study, “bad” *Aeromonas veronii* was described as causing uremic syndrome [29]. Among the potentially beneficial “toxin-decomposing” bacteria we identified *Lactobacillus casei*, *Lactobacillus rhamnosus* and *Lactobacillus acidophilus*, which were earlier described as a probiotic treatment for kidney failure. Despite the fact that none of them were common in HMP2 patients, their use as probiotics demonstrated positive effects [30,31,32,33,34,35]. *Helicobacter pylori*, which is conventionally considered to be a malignant bacterium, was also among the bacteria that can synthesize more toxins than they consume; however, there are no direct links between it and kidney diseases [36].

*Pediococcus acidilactici* is an excellent example of the limitations of the described approach. In our analysis, it was among both the synthesizing bacteria (it is able to synthesize five toxins without metabolizing them) and the toxin-metabolizing bacteria (six toxins). As mentioned above in the Results section, these are not the same toxins. The bacterium can synthesize homocysteine, indole-3-acetic acid, myoinositol, nicotinamide, and y-guanidinobutyric acid and can metabolize creatinine and cytidine methylglyoxal, S-adenosylhomocysteine, sorbitol, and xanthosine. Moreover, it can both synthesize and decompose nine more uremic retention solutes, which means that these solutes are intermediates in some metabolic pathways. For *Pediococcus acidilactici*, there exists a clinical trial where it was included in probiotic treatment for uremic patients, and thus, it could be defined as a “good” bacterium [37]. However, from these examples, one can easily see that it is quite possible to miss valid targets due to their scarcity in databases or a very broad range of accessible biochemical reactions for certain bacteria.

Moreover, it was found that the total presence of *Klebsiella* and *Escherichia coli* was significantly increased in the intestine of uremic patients [38]. Indeed, we identified these bacteria among those “metabolizing but not synthesizing” toxins. Using HMP2 data we have found, that Escherichia coli and Klebsiella pneumoniae are top-2 bacteria by number of detected enzymes’ mRNA which catalyze reactions with uremic retention solutes: 92 and 72 reactions for 31 and 30 different uremic toxins, accordingly. These findings perfectly aligns with mentioned study [38]. We expect such bacteria will benefit from an increase in uremic retention solute concentrations since they can utilize them during the reverse transport to the intestine from the blood. However, it is hard to distinguish the causality, i.e., whether the toxin increases because a bacterium produces it (and thus this is a “bad” bacterium) or whether the amount increases because it can utilize the toxin (and thus it is a “beneficial” one).

In this regard, it should be noted that bacteria can use uremic retention solutes as nutrients and thus eliminate hazardous solutes. For instance, some bacteria express urease (*Pseudomonas* spp.), the enzyme that catalyzes the hydrolysis of urea, or urate oxidase (*Clostridia* spp.), oxidizing uric acid [39]. Toxic substances such as oxalate and creatinine, when released to the gut, can also be subsequently metabolized by microbiota [40,41]. Microbiota species from the genera *Oxalobacter*, *Lactobacillus*, *Bifidobacterium*, *Enterococcus*, and *Eubacterium*, which are present in normal microbiota, are capable of degrading oxalate, hence diminishing its uremic accumulation [42]. According to our data, 39 bacterial species from the human microbiome are involved in compound breakdown.

We primarily focused on the microbiome’s effects on uremia development during our analysis. However, gut bacteria are also affected by uremic toxins. Uremia and chronic kidney disease alter the biochemical environment of the gut mainly through the entrance of urea into the intestine [43]. Uremic toxins are considered as a trigger for the systemic inflammation accompanying uremia [44]. A variety of conditions, including decreased pH and increased oxygen concentration, are observed in the intestinal lumen in CKD patients. Since the normal intestinal microbiota is sensitive to the milieu and predominantly consists of obligate anaerobes [45], such conditions are inappropriate for it. Hence, the normal symbiotic relationship between the microbiota and the host breaks down and results in dysbiosis [46]. The data regarding the total number of bacteria in healthy individuals and people with severe kidney disease show that the number of gut aerobes (*Enterobacteria*, *Enterococci*) is about 100 times higher in sick patients than in healthy ones, whereas the numbers of some anaerobes (*Bifidobacteria*) are decreased [38].

A comparison of microbial DNA isolated from the stools of healthy people and persons with end-stage renal disease (ESRD) reveals differences in 190 operational taxonomic units (OTUs) from 3 phyla and 13 families [43]. In general, the gut microbiota of CKD patients shows an abundance of pathogenic microflora, including some *Clostridia, Bacteroidia*, and *Gammaproteobacteris* (families *Enterobacteriaceae*, *Halomonadaceae*, *Moraxellaceae*, *Pseudomonadaceae*, *Thiotrichaceae*) [16,43,47]. These bacteria are able to survive in a transformed gut environment that has been changed due to the penetration of some uremic toxins into the gut. Some bacteria use uremic toxins as nutrients. For instance, urea could be utilized by urease, the enzyme that catalyzes the hydrolysis of urea (*Pseudomonas* spp.), and uric acid could be oxidized by urate oxidase (*Clostridia* spp.) [48]. Toxic substances such as oxalate and creatinine, when released to the gut, can be subsequently metabolized by changed microbiota also [40,49]. Thus, the uremic gut milieu works as a selective environment for microbiota resulting in uremia-associated biocenosis and, vice versa, uremic toxins can also be directly toxic to certain bacteria. For example, methylglyoxal is harmful to a number of microorganisms, both Gram-positive and Gram-negative species, since it reacts with the nucleophilic centers of macromolecules such as RNA, DNA, and proteins [50,51]. It has been suggested that, subsequently, the compound inhibits DNA replication and protein synthesis by interacting with the guanine residues of nucleic acids and their precursors [52]. In addition, methylglyoxal alters the formation of bacterial appendages, flagella, and fimbriae, resulting in impaired cell adherence and motility [53]. Nevertheless, some pathogenic species possess detoxification systems for this substance [54]. Probably, this is one of the major uremic toxins leading to a change in microflora under uremic conditions.

We also obtained a complete list of uremic toxins whose production is associated with the activity of various types of gut bacteria (Table 1). The relevance of the associations found for each solute is discussed below.

Trimethylamine-N-oxide and the closely related trimethylamine are well-known water-soluble uremic toxins. They are both considered to be microbiome-related [55]. Vanholder et al. estimated that they were among the most critical toxins due to demonstrated experimental and clinical toxic effects [56]. We found 141 human gut microbial species that theoretically contribute to trimethylamine-N-oxide production via two enzymatic reactions. On the other hand, mRNA for trimethylamine-N-oxide reductase, which can produce trimethylamine-N-oxide, was detected in the microbiota metatranscriptome of only 10% of patients (Table 4). This finding highlights the fact that bacterial enzyme abundance in a human microbiome is not an ideal predictor of the contribution to uremic toxin production. Apart from this, trimethylamine-N-oxide could be produced in the liver from trimethylamine by flavin monooxygenases, but since trimethylamine has a microbial origin the production of this and trimethylamine-N-oxide by the liver will at least in part depend on the intestinal microbiota. As well as renal failure, trimethylamine-N-oxide induces cardiovascular problems and stimulates upregulation of a variety of macrophage scavenger receptors related to atherosclerosis development [25]. However, the exact mechanism by which trimethylamine-N-oxide accumulation leads to atherosclerosis is still unproven [57]. Moreover, a link between increased levels of trimethylamine-N-oxide and the risk of a heart attack has been demonstrated [58]. Additionally, this compound is a suggested candidate for mediating type-2 diabetes mellitus [59,60].

3-(3-Hydroxyphenyl) propanoic acid was earlier described as being produced by the microbiome [61] and associated with schizophrenia and autism [62]. In our study, 103 bacteria were identified as able to produce this compound. Interestingly, this was again a uremic toxin produced by an enzyme that was present in only 10% of people in the HMP2 study (Table 4). However, in patients where this mRNA was detected, its quantity was in Q1 of all mRNA types in the transcriptome. Clinical observations in hemodialysis patients showed that this compound was reduced more than 10-fold in colectomy patients [15].

Another uremic toxin defined in our study as bacteria-derived, i.e., mannitol, is produced via a simple reaction in bacterial fructose and mannose metabolism. D-mannitol-1-phosphate phosphohydrolase catalyzes the hydrolysis of D-mannitol 1-phosphate to D-mannitol and phosphate. We found 227 gut bacteria species that produce the reaction. In contrast to trimethylamine-N-oxide and pseudouridine, mannitol can be further metabolized by several bacterial enzymes. However, the ratio of its synthesis to its metabolism is unknown. At the organism level, mannitol causes over-diuresis, with consequent dehydration [63]. Moreover, it demonstrates cytotoxicity to renal tubular epithelial cells, destroying the cell cytoskeleton [64].

We identified five specific bacterial enzymes that catalyze the production of phenol (an uremic solute with well-documented adverse effects in humans) causing protein denaturation with subsequent spreading necrosis [65]. Altogether, 192 gut bacterial species possess at least one of these enzymes. Phenol could be produced from tyrosine by microbial tyrosine phenol-lyase or may be synthesized from 4-hydroxybenzoate by 4-hydroxybenzoate decarboxylase or from catechol by phenol 2-monooxygenase, as parts of the aminobenzoate degradation pathway. The mRNA for three of these enzymes was found in 90% of people, and mRNA for arylesterase was in Q1 of all mRNA types for most patients.

Oxalate is a toxin enzymatically produced by bacteria, while humans synthesize it non-enzymatically. Oxalate is formed during purine metabolism when bacterial oxamate amidohydrolase catalyzes the transformation of oxamate to oxalate. We detected this reaction in 76 prokaryotic species from the human microbiome. In the HMP2 data, mRNA for oxalate-synthesizing enzymes was detected in the metatranscriptomes of 23% of patients, and all of these mRNA types were in Q1 of all mRNA types. As already mentioned, humans can produce oxalate non-enzymatically: for instance, ascorbic acid is metabolized in the human body with oxalate as an output [66]. Furthermore, significant amounts of oxalate enter the organism with food. The substance is known as the main component of kidney stones [67], and it modulates the immune system through an induced synthesis of cytokines, chemoattractants, and other inflammatory signal molecules, causing degradation of IκBα in proximal tubular cells [68]. It also has an unfavorable impact on mitochondrial function [69]. Note that the enzymes identified by database-based approaches need additional validation: oxalate and creatinine were regarded in this approach as “non-human origin” toxins, but they are produced in human organisms non-enzymatically, like pseudouridine, which is one of the main RNA catabolites [70,71,72].

**Figure 2:**
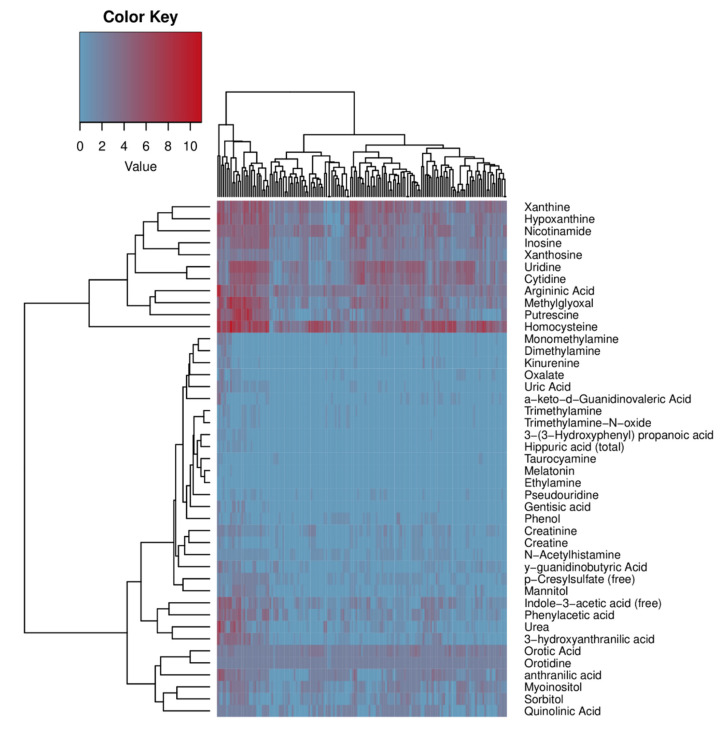
Cluster heatmap representing a clustering of toxins according to bacteria that can potentially synthesize them. Color represents the number of enzymatic reactions in given bacteria that can potentially lead to the synthesis of a toxin (see Supplementary Figure S1 for a high-resolution version with all bacteria names expanded).

In addition to toxins for which no potential synthetic pathways have been found in human metabolism, we showed that for several toxins there are only between one and three human enzymes that can potentially synthesize them. However, the question remains regarding whether these enzymes contribute significantly to toxins synthesis, i.e., how many of these enzymes there are in the organism. We suggested that an optional way to evaluate enzyme abundance in the whole organism was to use the Human Protein Atlas database, normalizing data on the mRNA abundance with respect to tissue weight. While understanding all the limitations of this approach, we believe that it can provide helpful information. Thus, we estimated that some of these human toxin-producing enzymes presented at a reasonable level in humans compared to others. Therefore, we propose that these enzymes have a low contribution to uremic retention solutes, and the role of the microbiota predominates in the synthesis of corresponding substances. For example, the orotidine concentration in the blood of uremic patients is quite high (1.20 (+/−1.60) mg/L), while the enzyme orotate phosphoribosyltransferase does not abound in human organisms (Figure 1). It is important to note that many uremic retention solutes are metabolically connected substances. One of the most important uremic retention solutes synthesis pathways is tryptophan metabolism through indolic and kynurenine pathways [73]. The following toxins are generated through this pathway: indoxyl sulfate (IS), indoxyl-β-d-glucuronide, and indole-3-acetic acid (IAA). Predictably, bacteria that have enzymes responsible for synthesizing one of the connected toxins usually have a whole pathway. It can be seen in Figure 2 that there is a noticeable clusterization between solutes of a similar chemical nature. For example, xanthosine, xanthine, hypoxanthine, and inosine, which are interconnected via one enzymatic reaction in the purine metabolism pathway, are clustered together via common bacteria species. This illustrates exactly the indicated phenomenon: toxins of a similar chemical nature are synthesized by the same types of bacteria.

### Limitations

The crucial limitation of this approach is database completeness. Not all uremic retention solutes were found in the KEGG database, enzymatic reactions were not always listed, and not all reaction directions (synthesis or degradation) were given. However, most of these databases are updated annually. We hope that it will be possible to include even more important details in the analysis, such as enzymatic reaction constants, bacteria abundance in the microbiome, and others.

## 4. Materials and Methods

### 4.1. Database Usage

The uremic toxins list was taken from the EUTox database (https://database.uremic-toxins.org/home.php, last accessed 1 December 2020) and contained 134 compounds. Additionally, 12 compounds were added after the literature analysis.

The Human Metabolome Database (https://hmdb.ca/, last accessed 1 December 2020) [22] was used to check the origins of toxins.

The human microbiome bacteria list was obtained from the NIH Human Microbiome Project (https://commonfund.nih.gov/hmp, last accessed 1 December 2020) [74]. Different strains were collapsed into a single data point for a bacterial species if possible. A list of 500 bacteria was obtained.

The Human Protein Atlas (https://www.proteinatlas.org/, last accessed 1 December 2020) was used to roughly estimate the abundance of enzymes in the human organism, using data on mRNA levels and average tissue weights.

The MetaCyc database (https://metacyc.org/, last accessed 1 December 2020) [75] was used to manually check the directions of the reactions if this information was not available in KEGG.

The Human Microbiome Project (HMP2) (https://ibdmdb.org/, last accessed 1 August 2021) [76] contains multi-omics data on the human microbiome in healthy and ill patients. 

### 4.2. Analysis

We analyzed 130 uremic toxins from the European Uremic Solutes Database (EUTox-DB) plus 12 from the literature analysis. For 97 toxins, we found identifiers in the KEGG database (release: 1 July 2020) [77].

The receipt and processing of information obtained from the KEGG database were carried out using the R package KEGGREST (version 1.26.1) [78].

A total of 69 toxins were involved in any KEGG reaction, and 54 toxins were involved in enzymatic reactions for which the metabolic pathway is known.

A total of 482 bacteria were identified in KEGG for which the genus, species, and strain (or only genus and species) corresponded to the bacteria from the HMP list. All enzymes involved in all metabolic pathways described for each bacterium from the list and separately for humans were obtained from the KEGG database.

We selected 67 enzymes that can metabolize uremic toxins and have a KEGG reaction ID and a KEGG EC ID. For each of the 67 enzymes, we received information about its identification at the protein (Proteomics section) and transcriptome (Metatranscriptomes section) levels by the donor or by bacteria, using HMP2 data.

All data manipulations and visualizations were performed using R version 3.6.3 and the packages tidyverse (version 1.3.1) and data.table (version 1.14.2).

### 4.3. Code Availability

All code used in the pipeline is available in GitHub at https://github.com/Zharikova/toxins, last accessed 24 October 2021.

## 5. Conclusions

We strongly recommend that our data and approach are not considered to be solid evidence of some bacteria being beneficial or malignant in conditions of uremia, but rather that they represent a tool for new insights during experimental analysis. We believe that even in limited form these approaches can contribute to the study of uremia-related bacteria and might contribute to the development of uremia treatment, suggesting new target bacteria and critical enzymatic reactions.

## Figures and Tables

**Table 1:** Potential synthesis and metabolism of uremic retention solutes by microbiome bacteria species and human organism.

Toxin	Number of Synthesizing or Metabolizing Bacteria Species	Number of Different Reactions in KEGG	Number of Enzymes in Human
Mannitol	56	4	0
Phenol	37	4	0
Trimethylamine	19	4	0
Oxalate	20	3	0
Creatinine	74	2	0
Trimethylamine-N-oxide	16	2	0
Pseudouridine	20	1	0
3-(3-Hydroxyphenyl) propanoic acid	13	1	0
S-Adenosylhomocysteine	143	35	1
Homocysteine	141	12	1
Argininic Acid	143	11	1
Putrescine	119	11	1
Methylglyoxal	138	10	1
Hypoxanthine	137	9	1
Urea	69	9	1
Xanthine	140	7	1
Nicotinamide	140	7	1
Cytidine	138	7	1
Uridine	138	7	1
anthranilic acid	108	7	1
Inosine	142	6	1
Indole-3-acetic acid	117	6	1
3-hydroxyanthranilic acid	70	6	1
a-keto-d-Guanidinovaleric Acid	27	6	1
Myoinositol	116	5	1
Phenylacetic acid	106	5	1
Sorbitol	94	5	1
Dimethylamine	15	5	1
Orotic Acid	143	4	1
Xanthosine	142	4	1
y-guanidinobutyric Acid	59	4	1
Monomethylamine	22	4	1
p-Cresyl sulfate	81	3	1
Uric Acid	34	3	1
Kinurenine	20	3	1
Orotidine	143	2	1
Quinolinic Acid	89	2	1
Creatine	44	2	1
Gentisic acid	12	2	1
N-Acetylhistamine	82	1	1
Hippuric acid	12	1	1
Taurocyamine	9	1	1
Melatonin	2	1	1
Ethylamine	1	1	1

**Table 2:** Bacteria determined as potential producers or consumers of some uremic retention solutes using analysis of KEGG modules with the MetaCyc database. Bacteria presented have more than five toxin-producing reactions (without decomposing the same toxin) or more than four toxin-decomposing reactions (without synthesizing the same toxin). A complete list is available in Supplementary Table S3. Bacteria identified as both synthesizing and decomposing are presented in bold.

KEGG + MetaCyc	
Synthesis (of More Than Five Toxins)	Decomposition (of More Than Four Toxins)
*Oxalobacter formigenes* ***Stenotrophomonas* sp.** *Campylobacter coli* *Brevundimonas* sp. *Campylobacter upsaliensis* *Geobacter* sp. ***Escherichia* sp.** ***Rhizobium* sp.** *Desulfovibrio* sp. *Methylobacterium* sp. *Dialister pneumosintes* *Helicobacter pylori* *Desulfovibrio piger* *Edwardsiella tarda* *Morganella morganii* *Gordonibacter pamelaeae* *Clostridium* sp. *Eggerthella lenta* *Coprococcus catus* ***Pediococcus acidilactici*** ***Lactobacillus fermentum*** ***Corynebacterium ammoniagenes*** *Desulfitobacterium hafniense* *Paenisporosarcina* sp. *Paenibacillus* sp. *Christensenella minuta* *Aeromonas veronii* *Propionibacterium* sp. *Helicobacter bilis* *Phascolarctobacterium faecium* *Lachnospiraceae bacterium* *Helicobacter cinaedi*	***Escherichia* sp.** ***Rhizobium* sp.** ***Pediococcus acidilactici*** *Lactobacillus ruminis* ***Stenotrophomonas* sp.** ***Lactobacillus fermentum*** *Lactobacillus paracasei* *Lactobacillus casei* *Lactobacillus rhamnosus* *Flavobacteriaceae bacterium* *Klebsiella* sp. *Lactobacillus amylolyticus* ***Corynebacterium ammoniagenes*** *Clostridium sporogenes* *Sphingomonas* sp. *Hafnia alvei* *Escherichia coli* *Providencia alcalifaciens* *Providencia rustigianii* *Enterobacter cloacae* *Listeria grayi* *Kocuria* sp. *Lactobacillus acidophilus* *Pseudomonas* sp. *Klebsiella oxytoca* *Lactobacillus helveticus*

**Table 3:** Uremic retention solutes and their synthesizing enzymes’ mRNA abundance in HMP2. Presented uremic retention solutes had at least one enzyme’s mRNA represented in more than 90% of patients. The following information is given in parentheses for each enzyme: mRNA - percentage of patients who have mRNA for this enzyme; Q - quartile for this mRNA abundance; prot - percent of patients who have protein for this enzyme detected in proteome. The complete list is available in Supplementary Table S5.

Uremic Retention Solutes	Number of Different Enzymes Where mRNA Detected	Percentage of Patients Who Have at Least One Enzyme’s mRNA for This Uremic Retention Solute	Enzymes
Argininic Acid	11	99.73%	Nitric-oxide synthase (NADPH) (mRNA: 2.59%, Q1, prot: NA); Glycine amidinotransferase (mRNA: 0.68%, Q1, prot: NA); Arginine N-succinyltransferase (mRNA: 5.17%, Q1, prot: NA); Arginine--pyruvate transaminase (mRNA: 0.14%, Q1, prot: NA); Arginine kinase (mRNA: 9.12%, Q2, prot: NA); [Protein ADP-ribosylarginine] hydrolase (mRNA: 2.86%, Q2, prot: NA); Arginase (mRNA: 82.31%, Q2, prot: 0.22%); Arginine deiminase (mRNA: 83.27%, Q2, prot: 8.22%); Arginine decarboxylase (mRNA: 91.29%, Q2, prot: 32%); Argininosuccinate lyase (mRNA: 99.18%, Q4, prot: 14%); Arginine--tRNA ligase (mRNA: 99.59%, Q4, prot: 42%)
S-Adenosylhomocysteine	64	99.59%	Homocysteine S-methyltransferase (mRNA: 4.49%, Q1, prot: NA); Protein-S-isoprenylcysteine O-methyltransferase (mRNA: 0%, QNA, prot: NA); Caffeoyl-CoA O-methyltransferase (mRNA: 8.98%, Q1, prot: NA); Uroporphyrinogen-III C-methyltransferase (mRNA: 52.65%, Q1, prot: 1.33%); Site-specific DNA-methyltransferase (cytosine-N(4)-specific) (mRNA: 2.45%, Q1, prot: NA); Precorrin-2 C(20)-methyltransferase (mRNA: 57.96%, Q1, prot: NA); Precorrin-3B C(17)-methyltransferase (mRNA: 78.91%, Q2, prot: NA); Precorrin-6B C(5,15)-methyltransferase (decarboxylating) (mRNA: 86.94%, Q2, prot: 0.22%); Precorrin-4 C(11)-methyltransferase (mRNA: 71.56%, Q1, prot: NA); Trans-aconitate 2-methyltransferase (mRNA: 8.98%, Q1, prot: 0.22%)... *(see all 64 enzymes in ST 4)*
Orotidine	2	99.59%	Orotate phosphoribosyltransferase (mRNA: 99.05%, Q4, prot: 35.78%); Orotidine-5’-phosphate decarboxylase (mRNA: 99.32%, Q4, prot: 63.78%)
Uridine	7	99.46%	Pyrimidine-nucleoside phosphorylase (mRNA: 50.88%, Q1, prot: 0.22%); Uridine phosphorylase (mRNA: 91.84%, Q2, prot: 4.67%); Uridine kinase (mRNA: 99.05%, Q4, prot: 2.89%); 5’-nucleotidase (mRNA: 98.64%, Q4, prot: 1.56%); 3’-nucleotidase (mRNA: 19.46%, Q1, prot: 0.89%); Uridine nucleosidase (mRNA: 0.54%, Q1, prot: NA); Cytidine deaminase (mRNA: 87.62%, Q2, prot: 11.56%)
Hypoxanthine	8	99.32%	Xanthine dehydrogenase (mRNA: 80%, Q2, prot: 0.89%); Purine-nucleoside phosphorylase (mRNA: 99.18%, Q4, prot: 62.67%); Thymidine phosphorylase (mRNA: 58.5%, Q1, prot: 0.89%); S-methyl-5’-thioinosine phosphorylase (mRNA: 0.41%, Q1, prot: NA); Hypoxanthine phosphoribosyltransferase (mRNA: 97.69%, Q3, prot: 6.89%); Purine nucleosidase (mRNA: 28.44%, Q1, prot: NA); Ribosylpyrimidine nucleosidase (mRNA: 3.67%, Q1, prot: NA); Adenine deaminase (mRNA: 96.46%, Q2, prot: 0.22%)
Cytidine	6	99.32%	Pyrimidine-nucleoside phosphorylase (mRNA: 50.88%, Q1, prot: 0.22%); Uridine kinase (mRNA: 99.05%, Q4, prot: 2.89%); 5’-nucleotidase (mRNA: 98.64%, Q4, prot: 1.56%); 3’-nucleotidase (mRNA: 19.46%, Q1, prot: 0.89%); Ribosylpyrimidine nucleosidase (mRNA: 3.67%, Q1, prot: NA); Cytidine deaminase (mRNA: 87.62%, Q2, prot: 11.56%)
Orotic Acid	4	99.32%	Dihydroorotate dehydrogenase (NAD(+)) (mRNA: 91.43%, Q3, prot: 14.44%); Dihydroorotate dehydrogenase (quinone) (mRNA: 93.47%, Q3, prot: 0.44%); Dihydroorotate oxidase (fumarate) (mRNA: 2.86%, Q1, prot: NA); Orotate phosphoribosyltransferase (mRNA: 99.05%, Q4, prot: 35.78%)
Nicotinamide	7	99.18%	Purine-nucleoside phosphorylase (mRNA: 99.18%, Q4, prot: 62.67%); Nicotinamide phosphoribosyltransferase (mRNA: 0.41%, Q1, prot: NA); NAD(+) ADP-ribosyltransferase (mRNA: 0.41%, Q2, prot: 0%); Purine nucleosidase (mRNA: 28.44%, Q1, prot: NA); Uridine nucleosidase (mRNA: 0.54%, Q1, prot: NA); ADP-ribosyl cyclase/cyclic ADP-ribose hydrolase (mRNA: 0%, QNA, prot: NA); Nicotinamidase (mRNA: 19.32%, Q1, prot: NA)
Xanthine	6	99.18%	Xanthine dehydrogenase (mRNA: 80%, Q2, prot: 0.89%); Purine-nucleoside phosphorylase (mRNA: 99.18%, Q4, prot: 62.67%); Xanthine phosphoribosyltransferase (mRNA: 98.37%, Q4, prot: 16.22%); Hypoxanthine phosphoribosyltransferase (mRNA: 97.69%, Q3, prot: 6.89%); Purine nucleosidase (mRNA: 28.44%, Q1, prot: NA); Guanine deaminase (mRNA: 42.04%, Q1, prot: 0%)
Inosine	6	99.18%	Purine-nucleoside phosphorylase (mRNA: 99.18%, Q4, prot: 62.67%); Inosine kinase (mRNA: 17.69%, Q1, prot: NA); 5’-nucleotidase (mRNA: 98.64%, Q4, prot: 1.56%); Purine nucleosidase (mRNA: 28.44%, Q1, prot: NA); Ribosylpyrimidine nucleosidase (mRNA: 3.67%, Q1, prot: NA); Adenosine deaminase (mRNA: 36.46%, Q1, prot: 0.22%)
Myoinositol	5	99.18%	Inositol 2-dehydrogenase (mRNA: 16.33%, Q1, prot: 0.89%); Galactinol--raffinose galactosyltransferase (mRNA: 0.27%, Q2, prot: NA); CDP-diacylglycerol--inositol 3-phosphatidyltransferase (mRNA: 3.54%, Q2, prot: NA); Inositol-phosphate phosphatase (mRNA: 87.48%, Q2, prot: 0.22%); Alpha-galactosidase (mRNA: 99.05%, Q3, prot: 11.56%)
Xanthosine	3	99.18%	Purine-nucleoside phosphorylase (mRNA: 99.18%, Q4, prot: 62.67%); 5’-nucleotidase (mRNA: 98.64%, Q4, prot: 1.56%); Purine nucleosidase (mRNA: 28.44%, Q1, prot: NA)
Methylglyoxal	8	99.05%	Aldehyde reductase (mRNA: 11.43%, Q1, prot: NA); Methylglyoxal reductase (NADPH) (mRNA: 5.71%, Q2, prot: NA); Glycerol dehydrogenase (mRNA: 54.01%, Q1, prot: 7.11%); Glyoxylate reductase (NADP(+)) (mRNA: 17.28%, Q1, prot: NA); Lactaldehyde dehydrogenase (mRNA: 24.76%, Q1, prot: 0.89%); D-lactate dehydratase (mRNA: 15.78%, Q1, prot: 0.22%); Methylglyoxal synthase (mRNA: 98.91%, Q4, prot: 58.22%); Lactoylglutathione lyase (mRNA: 66.8%, Q2, prot: 0.67%)
Homocysteine	8	99.05%	Homocysteine S-methyltransferase (mRNA: 4.49%, Q1, prot: NA); Methionine synthase (mRNA: 82.45%, Q2, prot: 0.44%); 5-methyltetrahydropteroyltriglutamate--homocysteine S-methyltransferase (mRNA: 38.23%, Q1, prot: 18.67%); Cystathionine gamma-synthase (mRNA: 29.52%, Q1, prot: 0.67%); O-acetylhomoserine aminocarboxypropyltransferase (mRNA: 94.01%, Q2, prot: 21.56%); Adenosylhomocysteinase (mRNA: 97.82%, Q3, prot: 16.22%); Cystathionine beta-synthase (mRNA: 4.35%, Q1, prot: NA); S-ribosylhomocysteine lyase (mRNA: 97.82%, Q3, prot: 42.89%)
Sorbitol	4	99.05%	L-iditol 2-dehydrogenase (mRNA: 63.27%, Q1, prot: 4.89%); Aldehyde reductase (mRNA: 11.43%, Q1, prot: NA); Hexokinase (mRNA: 33.88%, Q1, prot: NA); Alpha-galactosidase (mRNA: 99.05%, Q3, prot: 11.56%)
Phenylacetic acid	6	98.37%	Phenylacetaldehyde dehydrogenase (mRNA: 4.08%, Q1, prot: NA); Aldehyde dehydrogenase (NAD(P)(+)) (mRNA: 5.85%, Q1, prot: NA); Penicillin amidase (mRNA: 62.99%, Q1, prot: 0.44%); Amidase (mRNA: 1.09%, Q1, prot: NA); Nitrilase (mRNA: 0.27%, Q1, prot: NA); Phenylacetate--CoA ligase (mRNA: 98.23%, Q4, prot: 12.44%)
Putrescine	11	98.1%	Non-specific polyamine oxidase (mRNA: 0.41%, Q2, prot: NA); Putrescine carbamoyltransferase (mRNA: 1.5%, Q1, prot: NA); Diamine N-acetyltransferase (mRNA: 83.4%, Q2, prot: NA); Spermidine synthase (mRNA: 76.87%, Q2, prot: 0.44%); Homospermidine synthase (mRNA: 0.54%, Q1, prot: NA); Diamine transaminase (mRNA: 0.14%, Q1, prot: NA); Putrescine aminotransferase (mRNA: 16.19%, Q1, prot: NA); N-carbamoylputrescine amidase (mRNA: 28.84%, Q1, prot: NA); Agmatinase (mRNA: 74.29%, Q2, prot: 1.33%); Ornithine decarboxylase (mRNA: 48.71%, Q2, prot: 2.89%); Glutamate--putrescine ligase (mRNA: 6.8%, Q1, prot: NA)
Quinolinic Acid	2	98.1%	Nicotinate-nucleotide diphosphorylase (carboxylating) (mRNA: 77.55%, Q2, prot: 8.89%); Quinolinate synthase (mRNA: 97.82%, Q3, prot: 4.67%)
3-hydroxyanthranilic acid	4	97.82%	Catalase peroxidase (mRNA: 4.9%, Q2, prot: 5.11%); Catalase (mRNA: 97.69%, Q3, prot: 22.22%); 3-hydroxyanthranilate 3,4-dioxygenase (mRNA: 0.27%, Q2, prot: NA); Kynureninase (mRNA: 0.27%, Q2, prot: NA)
Urea	8	96.87%	Urease (mRNA: 58.78%, Q2, prot: 0.67%); Arginase (mRNA: 82.31%, Q2, prot: 0.22%); Agmatinase (mRNA: 74.29%, Q2, prot: 1.33%); Creatinase (mRNA: 0.95%, Q2, prot: NA); Allantoicase (mRNA: 0.68%, Q2, prot: NA); Guanidinobutyrase (mRNA: 0.27%, Q1, prot: NA); Ureidoglycolate lyase (mRNA: 5.44%, Q1, prot: 0.22%); Urea carboxylase (mRNA: 27.76%, Q1, prot: NA)
Anthranilic acid	5	96.33%	Anthranilate phosphoribosyltransferase (mRNA: 94.29%, Q2, prot: 2%); Arylformamidase (mRNA: 0.41%, Q1, prot: NA); Kynureninase (mRNA: 0.27%, Q2, prot: NA); Anthranilate synthase (mRNA: 55.1%, Q1, prot: 0.89%); Anthranilate--CoA ligase (mRNA: 0.27%, Q1, prot: NA)
Phenol	3	89.66%	Arylesterase (mRNA: 16.73%, Q1, prot: NA); 4-hydroxybenzoate decarboxylase (mRNA: 3.95%, Q2, prot: NA); Tyrosine phenol-lyase (mRNA: 86.67%, Q3, prot: NA)
Uric Acid	3	80.00%	FAD-dependent urate hydroxylase (mRNA: 0.68%, Q1, prot: NA); Xanthine dehydrogenase (mRNA: 80%, Q2, prot: 0.89%); 8-oxoguanine deaminase (mRNA: 0%, QNA, prot: NA)
Trimethylamine	3	78.23%	Betaine reductase (mRNA: 75.65%, Q3, prot: 0.44%); Trimethylamine dehydrogenase (mRNA: 0.27%, Q1, prot: NA); Trimethylamine-N-oxide reductase (cytochrome c) (mRNA: 10.2%, Q1, prot: 0.44%)
Hippuric acid	1	44.63%	Hippurate hydrolase (mRNA: 44.63%, Q1, prot: NA)
Monomethylamine	1	42.99%	Sarcosine reductase (mRNA: 42.99%, Q1, prot: 0.44%)
Creatine	3	28.44%	Creatine kinase (mRNA: 1.5%, Q1, prot: 0%); Creatininase (mRNA: 26.39%, Q1, prot: 0.67%); Creatinase (mRNA: 0.95%, Q2, prot: NA)
Creatinine	1	26.39%	Creatininase (mRNA: 26.39%, Q1, prot: 0.67%)
Oxalate	3	22.72%	Formyl-CoA transferase (mRNA: 20.54%, Q1, prot: 1.33%); CoA:oxalate CoA-transferase (mRNA: 3.27%, Q1, prot: 0.22%); Oxalate--CoA ligase (mRNA: 0.14%, Q1, prot: NA)
Mannitol	3	21.9%	D-arabinitol 4-dehydrogenase (mRNA: 0.68%, Q1, prot: NA); Mannitol dehydrogenase (mRNA: 0.41%, Q1, prot: NA); Mannitol 2-dehydrogenase (mRNA: 21.22%, Q1, prot: NA)
Phenol sulfate	2	20.27%	Aryl-sulfate sulfotransferase (mRNA: 5.99%, Q1, prot: NA); Arylsulfatase (mRNA: 15.37%, Q1, prot: NA)
Arab(in)itol	1	11.43%	Aldehyde reductase (mRNA: 11.43%, Q1, prot: NA)
Trimethylamine-N-oxide	1	10.2%	Trimethylamine-N-oxide reductase (cytochrome c) (mRNA: 10.2%, Q1, prot: 0.44%)
Pseudouridine	1	9.66%	Pseudouridine kinase (mRNA: 9.66%, Q1, prot: NA)
p-Cresyl sulfate	2	9.12%	4-hydroxyphenylacetate decarboxylase (mRNA: 0.95%, Q2, prot: NA); 2-iminoacetate synthase (mRNA: 8.71%, Q1, prot: NA)
3-(3-Hydroxyphenyl) propanoic acid	1	9.12%	3-(3-hydroxy-phenyl)propanoic acid hydroxylase (mRNA: 9.12%, Q1, prot: NA)
a-keto-d-Guanidinovaleric Acid	2	5.85%	D-amino-acid transaminase (mRNA: 5.71%, Q1, prot: NA); Arginine--pyruvate transaminase (mRNA: 0.14%, Q1, prot: NA)
Indole-3-acetic acid	3	3.95%	Aldehyde dehydrogenase (NAD(+)) (mRNA: 2.72%, Q1, prot: NA); Amidase (mRNA: 1.09%, Q1, prot: NA); Nitrilase (mRNA: 0.27%, Q1, prot: NA)
y-guanidinobutyric Acid	3	1.9%	Glycine amidinotransferase (mRNA: 0.68%, Q1, prot: NA); Amidase (mRNA: 1.09%, Q1, prot: NA); Guanidinobutyrase (mRNA: 0.27%, Q1, prot: NA)
Hyaluronic acid (Hyaluronan)	1	1.9%	Hyaluronan synthase (mRNA: 1.9%, Q1, prot: 0.22%)
Gentisic acid	1	1.22%	3-hydroxybenzoate 6-monooxygenase (mRNA: 1.22%, Q1, prot: 0.22%)
Kinurenine	4	0.95%	Kynurenine 3-monooxygenase (mRNA: 0%, QNA, prot: NA); Kynurenine--oxoglutarate transaminase (mRNA: 0.82%, Q2, prot: 0%); Arylformamidase (mRNA: 0.41%, Q1, prot: NA); Kynureninase (mRNA: 0.27%, Q2, prot: NA)
Dimethylamine	2	0.95%	Trimethylamine dehydrogenase (mRNA: 0.27%, Q1, prot: NA); Dimethylargininase (mRNA: 0.68%, Q2, prot: NA)
Kynurenic Acid	1	0.82%	Kynurenine--oxoglutarate transaminase (mRNA: 0.82%, Q2, prot: 0%)
Taurocyamine	1	0.68%	Glycine amidinotransferase (mRNA: 0.68%, Q1, prot: NA)
Asymmetric Dimethylarginine (ADMA)	1	0.68%	Dimethylargininase (mRNA: 0.68%, Q2, prot: NA)

**Table 4:** Occurrence and abundance of uremic-retention-solutes-synthesizing enzymes in patients. Presented enzymes were detected in more than 10% of patients. The full list is available in Supplementary Table S4.

Enzyme Name	Number of Bacteria That Express mRNA for This Enzyme	Percentage of Patients with Detected mRNA for This Enzyme	Quartile of mRNA Abundance	Percentage of Patients with Indicated Enzyme Detected in the Proteome	A Toxin That Could Be Potentially Synthesised by the Indicated Enzyme
Orotidine-5’-phosphate decarboxylase	205	99.32%	2	63.78%	Orotidine
Purine-nucleoside phosphorylase	187	99.18%	2	62.67%	Hypoxanthine; Inosine; Nicotinamide; Xanthine; Xanthosine
Methylglyoxal synthase	124	98.91%	3	58.22%	Methylglyoxal
S-ribosylhomocysteine lyase	129	97.82%	3	42.89%	Homocysteine
Arginine--tRNA ligase	221	99.59%	2	42%	Argininic Acid
Orotate phosphoribosyltransferase	199	99.05%	3	35.78%	Orotic Acid; Orotidine
Arginine decarboxylase	39	91.29%	2	32%	Argininic Acid
Catalase	56	97.69%	3	22.22%	3-hydroxyanthranilic acid
O-acetylhomoserine aminocarboxypropyl-transferase	46	94.01%	2	21.56%	Homocysteine
5-methyltetrahydropteroyltriglutamate-homocysteine S-methyltransferase	31	38.23%	1	18.67%	Homocysteine
Xanthine phosphoribosyltransferase	161	98.37%	2	16.22%	Xanthine
Adenosylhomocysteinase	68	97.82%	2	16.22%	Homocysteine; S-Adenosylhomocysteine
Dihydroorotate dehydrogenase (NAD(+))	28	91.43%	2	14.44%	Orotic Acid
Argininosuccinate lyase	192	99.18%	2	14%	Argininic Acid
Phenylacetate--CoA ligase	84	98.23%	2	12.44%	Phenylacetic acid
tRNA-2-methylthio-N(6)-dimethylallyladenosine synthase	185	99.05%	2	11.56%	S-Adenosylhomocysteine
Alpha-galactosidase	71	99.05%	2	11.56%	Myoinositol; Sorbitol
Cytidine deaminase	48	87.62%	3	11.56%	Cytidine; Uridine

**Table 5:** Top 20 (in terms of occurrence) bacteria that metabolize uremic retention solutes.

Bacteria	Percentage of Patients with Detected mRNA for Indicated Bacteria	Number of Uremic Retention Solutes for Which mRNA Was Detected of at Least One of the Toxin-Producing Enzymes	Number of Different Enzymes Detected for Indicated Bacteria	Uremic Retention Solutes That Could Be Potentially Synthesized by Indicated Bacteria
*Ruminococcus torques*	93.61%	22	45	Sorbitol; Arab(in)itol; Methylglyoxal; Hypoxanthine; Uric Acid; Xanthine; S-Adenosylhomocysteine; Homocysteine; Inosine; Nicotinamide; Xanthosine; Orotic Acid; Orotidine; anthranilic acid; Uridine; Putrescine; Quinolinic Acid; Cytidine; Myoinositol; Urea; Argininic Acid; Phenylacetic acid
*Faecalibacterium prausnitzii*	93.33%	17	34	Sorbitol; Homocysteine; S-Adenosylhomocysteine; Hypoxanthine; Inosine; Nicotinamide; Xanthine; Xanthosine; Orotic Acid; Orotidine; Putrescine; Cytidine; Uridine; Myoinositol; Urea; Argininic Acid; Methylglyoxal
*Eubacterium rectale*	81.77%	17	27	Sorbitol; S-Adenosylhomocysteine; Hypoxanthine; Inosine; Nicotinamide; Xanthine; Xanthosine; Orotic Acid; Orotidine; anthranilic acid; Uridine; Putrescine; Homocysteine; Quinolinic Acid; Cytidine; Methylglyoxal; Argininic Acid
*Bacteroides uniformis*	79.05%	18	20	S-Adenosylhomocysteine; Hypoxanthine; Inosine; Nicotinamide; Xanthine; Xanthosine; Orotic Acid; Orotidine; anthranilic acid; Quinolinic Acid; Cytidine; Uridine; Myoinositol; Sorbitol; Methylglyoxal; Argininic Acid; Homocysteine; Phenylacetic acid
*Bacteroides ovatus*	78.37%	21	34	Sorbitol; Mannitol; 3-hydroxyanthranilic acid; Orotic Acid; S-Adenosylhomocysteine; Putrescine; Hypoxanthine; Inosine; Nicotinamide; Xanthine; Xanthosine; Orotidine; anthranilic acid; Homocysteine; Quinolinic Acid; Cytidine; Uridine; Myoinositol; Phenylacetic acid; Methylglyoxal; Argininic Acid
*Bacteroides vulgatus*	77.14%	17	24	Orotic Acid; S-Adenosylhomocysteine; Hypoxanthine; Inosine; Nicotinamide; Xanthine; Xanthosine; Orotidine; anthranilic acid; Cytidine; Uridine; Myoinositol; Sorbitol; Methylglyoxal; Argininic Acid; Homocysteine; Phenylacetic acid
*Ruminococcus obeum*	75.78%	23	41	Sorbitol; Arab(in)itol; Methylglyoxal; Hypoxanthine; Uric Acid; Xanthine; S-Adenosylhomocysteine; Inosine; Nicotinamide; Xanthosine; Orotic Acid; Orotidine; anthranilic acid; Quinolinic Acid; Uridine; Putrescine; Homocysteine; Cytidine; Phenol sulfate; Phenol; Urea; Argininic Acid; Phenylacetic acid
*Lachnospiraceae bacterium*	68.98%	13	16	S-Adenosylhomocysteine; Hypoxanthine; Inosine; Nicotinamide; Xanthine; Xanthosine; Orotic Acid; Orotidine; Cytidine; Uridine; Homocysteine; Argininic Acid; Urea
*Roseburia inulinivorans*	62.04%	15	21	S-Adenosylhomocysteine; Hypoxanthine; Inosine; Nicotinamide; Xanthine; Xanthosine; Orotic Acid; Orotidine; Uridine; Putrescine; Quinolinic Acid; Cytidine; Methylglyoxal; Argininic Acid; Homocysteine
*Bacteroides thetaiotaomicron*	61.77%	19	30	3-hydroxyanthranilic acid; Orotic Acid; S-Adenosylhomocysteine; Hypoxanthine; Inosine; Nicotinamide; Xanthine; Xanthosine; Orotidine; anthranilic acid; Homocysteine; Quinolinic Acid; Cytidine; Uridine; Myoinositol; Sorbitol; Methylglyoxal; Argininic Acid; Phenylacetic acid
*Clostridium bolteae*	61.5%	16	22	Sorbitol; S-Adenosylhomocysteine; Hypoxanthine; Inosine; Nicotinamide; Xanthine; Xanthosine; Orotic Acid; Orotidine; anthranilic acid; Uridine; Quinolinic Acid; Methylglyoxal; Argininic Acid; Homocysteine; Phenylacetic acid
*Bacteroides caccae*	60.95%	18	26	Orotic Acid; S-Adenosylhomocysteine; Hypoxanthine; Inosine; Nicotinamide; Xanthine; Xanthosine; Orotidine; anthranilic acid; Homocysteine; Quinolinic Acid; Cytidine; Uridine; Myoinositol; Sorbitol; Methylglyoxal; Argininic Acid; Phenylacetic acid
*Bacteroides xylanisolvens*	60.14%	21	37	Sorbitol; Mannitol; Orotic Acid; S-Adenosylhomocysteine; Putrescine; Hypoxanthine; Inosine; Nicotinamide; Xanthine; Xanthosine; Orotidine; anthranilic acid; Homocysteine; Quinolinic Acid; Cytidine; Uridine; Myoinositol; Phenol sulfate; Phenylacetic acid; Methylglyoxal; Argininic Acid
*Dorea longicatena*	59.32%	16	25	Sorbitol; Methylglyoxal; Homocysteine; S-Adenosylhomocysteine; Hypoxanthine; Inosine; Nicotinamide; Xanthine; Xanthosine; Orotic Acid; Orotidine; Uridine; Quinolinic Acid; Myoinositol; Argininic Acid; Phenylacetic acid
*Roseburia hominis*	59.32%	16	21	S-Adenosylhomocysteine; Hypoxanthine; Inosine; Nicotinamide; Xanthine; Xanthosine; Orotic Acid; Orotidine; anthranilic acid; Putrescine; Quinolinic Acid; Cytidine; Uridine; Methylglyoxal; Argininic Acid; Homocysteine
*Roseburia intestinalis*	57.14%	20	42	Sorbitol; Hypoxanthine; Uric Acid; Xanthine; S-Adenosylhomocysteine; Inosine; Nicotinamide; Xanthosine; Orotic Acid; Orotidine; anthranilic acid; Quinolinic Acid; Cytidine; Uridine; Putrescine; Homocysteine; Phenol; Myoinositol; Argininic Acid; Methylglyoxal
*Parabacteroides merdae*	56.05%	17	23	Orotic Acid; S-Adenosylhomocysteine; Hypoxanthine; Inosine; Nicotinamide; Xanthine; Xanthosine; Orotidine; anthranilic acid; Quinolinic Acid; Cytidine; Uridine; Homocysteine; Argininic Acid; Urea; Methylglyoxal; Phenylacetic acid
*Bifidobacterium longum*	55.1%	16	21	S-Adenosylhomocysteine; Orotic Acid; Orotidine; anthranilic acid; Xanthine; Hypoxanthine; Putrescine; Homocysteine; Cytidine; Inosine; Uridine; Xanthosine; Myoinositol; Sorbitol; Nicotinamide; Argininic Acid
*Flavonifractor plautii*	54.83%	13	17	S-Adenosylhomocysteine; Hypoxanthine; Inosine; Nicotinamide; Xanthine; Xanthosine; Orotic Acid; Orotidine; Cytidine; Uridine; Homocysteine; Argininic Acid; Urea
*Alistipes putredinis*	54.56%	15	19	3-hydroxyanthranilic acid; Homocysteine; S-Adenosylhomocysteine; Hypoxanthine; Inosine; Nicotinamide; Xanthine; Xanthosine; Orotic Acid; Orotidine; Quinolinic Acid; Cytidine; Uridine; Methylglyoxal; Argininic Acid

## Supplementary Materials

The following are available online at https://www.mdpi.com/article/10.3390/ijms23010483/s1. Figure S1: Expanded cluster heatmap, which represents a clustering of toxins by bacteria that can potentially synthesize them. Table S1: All found toxin-reaction–bacteria connections. Table S2: Aggregated data on toxin-reaction–bacteria connections. Table S3: Full aggregated data on bacteria’s ability to synthesize or metabolize toxins. Table S4: List of toxins synthesized or metabolized by each bacterium. Table S5: Analysis of the presence of mRNA and proteins of potential uremic-toxins-synthesizing enzymes in metatranscriptomic and proteomic data from Human Microbiome Project database.
