# Supplementary figures and images for: Gut Microbiota as a Source of Uremic Toxins
Source: Int J Mol Sci. 2022 Jan 1;23(1):483. doi: 10.3390/ijms23010483 (PMC8745165; doi:10.3390/ijms23010483)

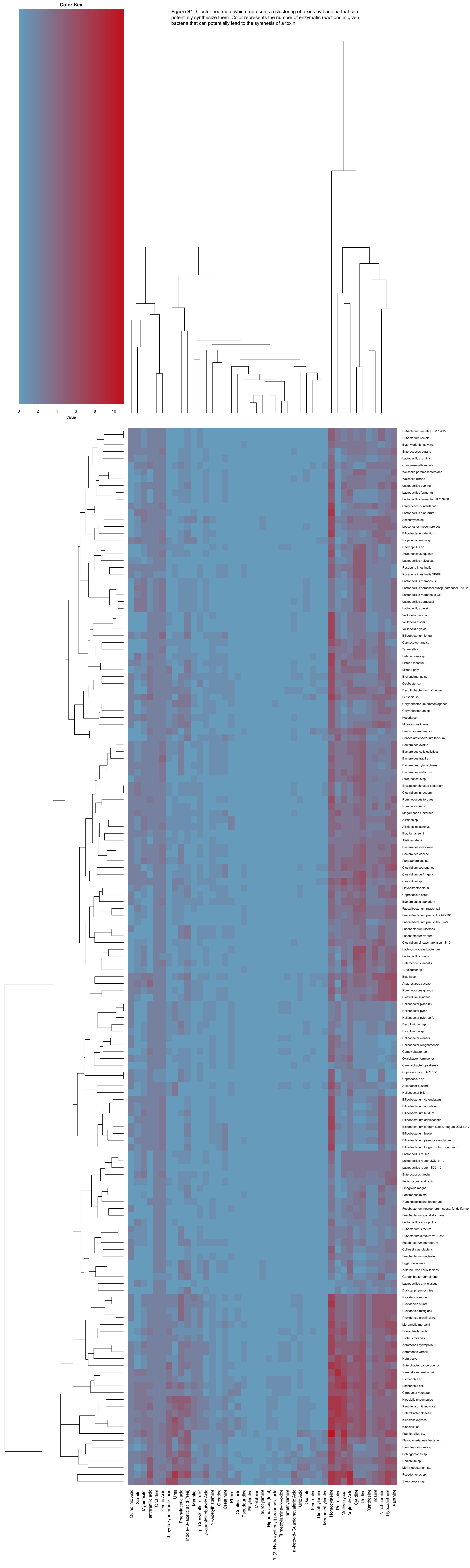

Supplement: Supplementary file 1 [file ijms-23-00483-s001.zip › sup/FS1.pdf]
